# PHYSICIANS’ KNOWLEDGE ABOUT PATIENTS’ RELIGIOUS BELIEFS IN PEDIATRIC CARE

**DOI:** 10.1590/1984-0462/;2019;37;4;00003

**Published:** 2019-06-19

**Authors:** Lucas Zambusi Naufel, Maíra Terra Cunha Di Sarno, Maria Augusta Junqueira Alves

**Affiliations:** a Irmandade da Santa Casa de Misericórdia de São Paulo, São Paulo, Brazil.

**Keywords:** Religion, Spirituality, Palliative care, Child, Religião, Espiritualidade, Cuidados paliativos, Criança

## Abstract

**Objective:**

To describe the knowledge of pediatricians and pediatric residents about the meaning of death according to the most prevalent religions in Brazil.

**Methods:**

A cross-sectional survey was conducted among pediatricians and pediatric residents at a tertiary-level children’s hospital in the city of São Paulo, SP, Brazil, questioning about their knowledge and experience related to spiritual care and the most common religious beliefs among pediatric palliative care patients in Brazil.

**Results:**

116 physicians answered the questionnaire, 98 (84.5%) considered themselves religious, defined as followers of any spiritual creed around the world, and 18 (15.5%) non-religious. Of the total, 97 (83.6%) considered themselves capable of dealing with the spiritual care of Catholic patients, 49 (42.2%) of Protestant patients and 92 (79.3%) of patients that follow Spiritism in the process of death. Religious doctors used less chaplaincy services than non-religious doctors (relative risk - RR 2.54; p=0.0432; confidence interval of 95% - 95%CI 1.21-5.34). Among the physicians, 111 (96%) believe that spirituality is beneficial in accepting the death process, responses were associated with the religiosity of the physicians (RR 1.18; p=0.0261; 95%CI 0.95-1.45). Also, 106 (91.4%) are unaware of the religion of their patients and the same number of participants consider pediatricians, in general, unprepared to deal with the spiritual aspect of death. These data are not associated with the participants’ religiosity.

**Conclusions:**

Although most pediatricians and residents consider themselves able to deal with the most prevalent religions in Brazil and affirm that spirituality is beneficial during the death process, little importance is given to the spiritual identity of their patients, which could limit an appropriate approach to their death process.

## RESUMO

**Objetivo:**

Descrever o conhecimento dos médicos pediatras e residentes de pediatria a respeito do significado da morte segundo as religiões mais prevalentes no Brasil.

**Métodos:**

Estudo transversal em que foi aplicado um questionário aos médicos pediatras e residentes de um hospital pediátrico de nível terciário da cidade de São Paulo, SP, Brasil, sobre o conhecimento e as experiências acerca de religiões de pacientes paliativos.

**Resultados:**

Ao todo, 116 médicos responderam ao questionário, 98 (84,5%) religiosos, definidos como seguidores de algum dogma espiritual existente no mundo, e 18 (15,5%) não religiosos. Do total, 97 (83,6%) considera-se apto a lidar com os cuidados espirituais de pacientes católicos, 49 (42,2%) de pacientes protestantes e 92 (79,3%) de pacientes espíritas em processo de morte. Médicos religiosos utilizaram menos os serviços de capelania do que médicos não-religiosos (risco relativo - RR 2,54; p=0,043; intervalo de confiança de 95% - IC95% 1,21-5,34). Dos entrevistados, 111 (96%) acreditam que a espiritualidade é benéfica na aceitação do processo de morte, resposta associada à religiosidade dos médicos (RR 1,18; p=0,026; IC95% 0,95-1,45). Ainda, 106 (91,4%) dos entrevistados desconhecem a religião de seus pacientes. A mesma quantidade de participantes considera os pediatras, em geral, despreparados para lidar com o aspecto espiritual da morte, e esses dados não estão ligados à manifestação de religiosidade.

**Conclusões:**

Apesar de a maioria dos médicos pediatras e residentes se dizer apta a lidar com as religiões mais prevalentes no Brasil e afirmar que a espiritualidade é benéfica durante o processo de morte, pouca importância é dada à identidade espiritual de seus pacientes, o que pode dificultar uma abordagem adequada ao seu processo de morte.

## INTRODUCTION

Even though death is inevitable for any living creature, man is the only one with a real awareness of his own finitude. It shapes our way of life and the way we see our own death: is there another form of consciousness? Is there eternal rest in paradisiacal fields? Will we reincarnate into new bodies? Or is there simply no more biological activity? Mixed with our doubts and longing for answers, religions and their philosophies are an important part of the dying process.

Objectively and for documentation purposes, medicine follows definitions that are prescribed and rigid in order to establish the death of an individual. Those definitions will not be discussed here, because the focus of this work is not on diseases, but people. The context for this study is the philosophical-existential questions that permeate the end of pediatric patients’ lives. Its purpose is to provide the patients (and their family) with physical, emotional, psychological, social and spiritual support, even post mortem.^1^

Current technological advances extend lives through medicine. However, despite lots of effort, they have a limited prognosis. Due to such prolongation in the dying process, there is a need for improvements in the implementation of pediatric palliative care, which is provided by multidisciplinary teams. They are responsible for the comprehensive and humanized care of individuals, encompassing the spheres of physical, emotional, social and spiritual pain,^2^ in order to promote the quality of life of and relieve the suffering of the patient and their family.^3^

Among the four spheres of palliative care offered to patients, the present study emphasizes the spiritual sphere, since there is a certain lack of medical preparation in dealing with patients’ individual beliefs and existential convictions. In spite of having a specialist degree in palliative care, knowledge about spirituality leverages the doctor-patient relationship and substantially alters how the child and his or her family will go through the dying process.^4^ ^.^ ^5^

The role of the chaplain is emphasized in the context of the pediatric palliative care teams’ multidisciplinary approach. After the enactment of Law No. 9.982, in 2000, religious assistance was granted to public and private hospitals. Representatives of various religions were given open access to these establishments.^6^ Chaplains were then added to the palliative care teams. In this role, their main function is to offer spiritual, emotional and social support, taking into account and respecting the singularities and subjectivities of the patients, their caregivers and the health professionals themselves.^7^

With this advancement, specialized care has been made to avoid ethical conflicts between the professional team and its patients.^8^ But in order for this to happen, doctors, as the leaders of the multidisciplinary teams, must understand the main tenets of each religion. Considering this responsibility and the plurality of religious beliefs in Brazilian culture, we questioned whether there are enough pediatric doctors and residents in the field of Brazilian pediatrics to deal with the spiritual aspect of their patients’ dying process.

## METHOD

This is a cross-sectional study in which a questionnaire was applied to pediatricians and pediatric residents of a large tertiary, philanthropic and secular pediatric hospital in the city of São Paulo, Brazil. These professionals, who work in emergency rooms, Intensive Care Units (ICUs) and pediatric, clinical and oncological infirmaries, were asked about their knowledge and experience with regard to palliative patients’ beliefs and religions.

We included all individuals who were duly enrolled in the medical residency program in addition to doctors contracted by the research hospital in 2017, and who signed the informed consent form. Those who were not part of the clinic, who did not present themselves with an active registration in the medical residency program, or who did not sign the informed consent form were excluded.

The terms *spirituality* and *religiosity* were used as synonyms throughout our study. They are defined as dogmas and thoughts that belong to some of the existing religions in the world.

A questionnaire was developed and applied with an informed consent form, which was written and reviewed by the responsible parties of this study. There was no validation or psychometric assessment. Through this questionnaire, the gender, age, undergraduate degree and field of each participant were identified.

The questionnaire was composed of 22 closed questions with “yes” or “no” answers about participants’ knowledge and experience regarding pediatric patients’ religions. A request to participate in the survey was made once, in person, and the questionnaires were completed in person and in writing, immediately (Table 1).

**Table 1:** Questionnaire given to the participants.

Questions*
1. Do you have any religion?
2. If so, which one?
3. Do you know the meaning of death according to your religion?
4. Do you know the fate of individuals after their death according to your religion?
5. Do you know the religion of all of your patients?
6. Do you think religion often helps patients and their families “accept” the dying process or “reject” it?
7. Do you believe that religion relieves the child and their family’s suffering in palliative care?
8. Have you requested help from the chaplaincy service at the hospital where you work?
9. Do you know the meaning of death according to Catholicism?
10. Do you know the meaning of death according to Protestantism?
11. Do you know the meaning of death according to Spiritism?
12. Do you know the meaning of death according to Umbanda?
13. Do you know the meaning of death according to Jehovah’s Witness?
14. Do you know the meaning of death according to Judaism?
15. Would you know how to comfort a Catholic patient using your knowledge of religion and going beyond explaining about death and one’s fate after death?
16. Would you know how to comfort a Protestant patient using your knowledge of religion and going beyond explaining about death and one’s fate after death?
17. Would you know how to comfort a Spiritist patient using your knowledge of religion and going beyond explaining about death and one’s fate after death?
18. Would you know how to comfort a Umbanda patient using your knowledge of religion and going beyond explaining about death and one’s fate after death?
19. Would you know how to comfort a Jehovah’s Witness patient using your knowledge of religion and going beyond explaining about death and one’s fate after death?
20. Would you know how to comfort a Jewish patient using your knowledge of religion and going beyond explaining about death and one’s fate after death?
21. Do you think pediatricians and pediatric residents, in general, are prepared to deal with the spiritual aspect of the death of their patients?
22. In your opinion, do you think that a course that teaches about different religions and how to approach them should be instituted in medical undergraduate programs?

*The possible answers were “yes” or “no”, except for question 2, whose alternatives were the six religions studied and an “other” option, with an open field for a response; and question 6, whose alternatives were “accept” or “reject.”

Included in this questionnaire were the five most prevalent religions in Brazil - Catholicism, Protestantism, Spiritism, Jehovah’s Witness and Umbanda - as well as Judaism, which, despite being the ninth most common religion in the country, is very frequent in the community studied.

Qualitative variables are presented in the frequencies and percentages, and quantitative variables are present in the summary measures. To evaluate the association between qualitative variables, the chi-square and Fisher’s exact tests were used. The level of significance was 5%. Statistical Package for the Social Sciences (SPSS) version 13.0 was used for analysis.

Approval for conducting this research was obtained from the Committee of Ethics in Research of the School of Medical Sciences at Santa Casa de Misericórdia de São Paulo.

## RESULTS

252 members of the institution’s clinical staff, including doctors and residents, were invited. Of these, 116 (46%) people answered the questionnaire. 97 (84%) were women, 18 (16%) were men and one did not identify. All of the questionnaires were fit for analysis. The mean age of all of the participants was 31.4 years old, with a median age of 28 years old. Among the participants, 61 (53%) individuals were doctors in their first or second year of general pediatric residency, 29 (25%) were residents of pediatric specialties, and 26 (22%) had already completed their pediatric specialization.

Of the 116 respondents, 106 (94%) said they did not know the religion of all the patients they attended. 111 (96%) believed that having a religion helped patients accept the dying process. 115 (99%) thought that religion helps provide relief to the patient and their relatives, and 21 (18%) had already made use of the chaplaincy service at their hospital.

Of the participants, 98 (85%) claimed to have a religion. 69 (60%) were Catholic, 18 (16%) were Spiritists, 6 were Protestant (5%) and there were no Umbanda or Jehovah’s Witness participants. Although Judaism was included in our study, only two participants (2%) were Jews, and three were classified as “other religions,” two (2%) Christian and one (1%) spiritualist religion, and 18 (15%) affirmed that they did not have any religion.

Of all the interviewees, 108 (93%) reported knowing about death in Catholicism, 49 (42%) in Protestantism, 92 (79.3%) in Spiritism, 22 (19%) in Umbanda, 14 (12%) in Jehovah’s Witness and 28 (24%) in Judaism.

When asked if they could use their knowledge to explain the dying process and comfort patients according to each religion, 97 (84%) said they were prepared to deal with Catholicism, 48 (41%) with Protestantism, 70 60%) with Spiritism, 15 (13%) with Umbanda, 12 (10%) with Jehovah’s Witness, and 22 (19%) with Judaism.

Of those interviewed, 106 (91%) answered that pediatricians and pediatric residents are not able to deal with the spiritual aspect of their dying patients, and 88 (76%) participants thought a course on religions should be instituted in Brazilian medical undergraduate programs.

The statistical analyzes below describe the significance and strength of the association (relative risk - RR) between the variables “physician’s religiosity” (present or absent), “sex” (male or female) and “professional category” medical residents or pediatricians) and the questions (Q) addressed in our questionnaire.

Regarding the physician’s religiosity, there was a significant association of responses with questions 6, 8, 9, 14, 15 and 22:

- Q6: 96% (111/116) of physicians asserted that religion helps accept death. Of the total, 98% (96/98) of physicians who declared themselves to be religious responded affirmatively to this question versus 83.3% (15/18) of physicians who declared themselves to be non-religious (RR 1.18; p=0.026, 95% confidence interval - 95%CI 0.95-1.45).
- Q8: 15% (15/98) of the doctors who declared themselves to be religious used the chaplaincy service versus 39% (7/18) of the doctors who declared themselves to be nonreligious (RR 2.54; p=0.043, 1.21-5.34).
- Q9: There was an association among the religious doctors who answered affirmatively to know about the dying process in Catholicism. Of the total, 97% of the doctors (95/98) who declared themselves to be religious answered affirmatively to know about the dying process in Catholicism versus 72% (13/18) of doctors who declared themselves to be nonreligious (RR 1.34; p=0.022, 95%CI 1.01-1.79).
- Q14: 19.4% of doctors (19/98) who declared themselves to be religious answered affirmatively to knowing about the dying process in Judaism versus 50% (9/18) of doctors who declared themselves to be non-religious (RR 2.58; p=0.013; 95%CI 1.40-4.76).
- Q15: 89% (87/98) of physicians who declared themselves to be religious answered affirmatively regarding knowing how to provide comfort in the death of Catholic patients versus 1668 56% (10/18) of physicians who declared themselves to be nonreligious (RR 1.60, p=0.020; 95%CI 1.05-2.43).
- Q22: 81% (79/98) of physicians who declared themselves to be religious stated that it is necessary to implement religious education during undergraduate programs versus 50% (9/18) of physicians who declared themselves to be nonreligious (RR 1.61; p=0.013; 95%CI 1.01-2.59).

Regarding the gender of the physicians, there was a significant association of responses with questions 14 and 20:

- Q14: 20% (19/97) of the female physicians answered affirmatively to know about the dying process in Judaism versus 44% (8/18) of male physicians (RR 2.27; p=0.033, 95%CI, 1.18-4.37).
- Q20: 16% (15/97) of female physicians answered affirmatively to know how to provide comfort in the death of Jewish patients versus 39% (7/18) of male physicians (RR 2.52; p=0.044; 95%CI 1, 20-5, 29).

With regard to resident physician *versus* professional doctor, there was a significant association of the responses only on question 8:

- Q8: 10% (9/90) of resident physicians used the chaplaincy service *versus* 46 (12/26) of professional physicians (RR 4.61; p<0.001, 95%CI 2.19-9.73).

## DISCUSSION

In Brazil, because of its history of colonization and Latin American culture, religiosity, especially Catholicism, is intimately tied to the daily life of the population. It should be noted, however, that religion is more than a set of visions and practices of a people and that its value cannot be simplified, for it offers individuals a spiritual way of living in this world.^9^

Although Catholicism is prevalent in Brazil and the medical literature tends to make more references to Christian religions in the research, a great diversity of religions among Brazilians is highlighted, and it is possible for pediatricians to encounter each one of them in clinical practice.

In the case of pediatric palliative care, one does not deal only with the patient, but also with his or her family. The dying process for a child, even if he or she was born with diseases that have reserved prognoses, it is an unexpected event for their relatives, as it will always be considered outside the “natural order.” Therefore, a family-centered care system should benefit both the patient and their relatives.^10^

In this encounter, the six priorities that parents seek to find in the end-of-life care of their children are evident: honest and complete information, quick access to the medical team, coordination of communication and care, expressing emotions and receiving support from the medical team, preservation of the integrity of the relationship between parents and children, and support from religion through faith in God.^11^

Scholars have noted that parents and children who have received comprehensive palliative care had faith as an important aid during the dying process, helping them understand situations and accept procedures that would not prolong the child’s life unnecessarily.^12^

In our research, most of the doctors (93%) said they knew about the meaning of death according to Catholicism, but this number is reduced to 84% when questioned if they feel prepared to cope with this information when dealing with patients in the dying process and their families. The same findings were repeated with the other less prevalent religions, with higher rates of knowledge about the meaning of death compared to their readiness to approach it, suggesting doctors’ insecurities and lack of knowledge about religious views.

One of the most relevant data of the present study is that among the 116 interviewees, 91% reported *not* knowing the religion of the patients they treated, showing how, starting from the initial anamnesis and identification, little interest is given to this aspect in clinical practice and medical education. In addition, 91% of the respondents are of the opinion that pediatricians and pediatric residents are generally unable to cope with the religious aspects of their patients.

Thus, although our study does not subjectively analyze the knowledge of each participant, because it is limited to self-assessment through an applied form, we questioned the importance of having a course geared toward the teaching of religions in medical undergraduate programs and how to approach it. This could expand physicians’ views about their patients and increase their adherence to the spiritual care of their patients. Most of the participants (75.9%) said they were in favor of it, with more support among doctors who were already religious. Considering this context, it is noted that the American Academy of Pediatrics proposes guidelines to include measures to help medical students in their practicums. These measures would help them understand the importance of preparing for palliative care, including with regard to religion, which, although it is relevant in consoling patients and their families and creating ties between relatives and caregivers, is often overlooked by health professionals.^13^

It is also noteworthy that only 18% of the participants had already started using the chaplaincy service in their hospitals. And it was even less common among residents and religious doctors, perhaps because it had been around less time and there were fewer opportunities in the first group and more confidence about religious knowledge in the second group. However, our data does not allow us to go deeper into this discussion.

In a study of hospitalized adults,^14^ 77% of the participants reported that physicians should consider the spiritual aspects of their patients and at least one third (37%) of the patients wanted their beliefs to be discussed more often. 68% reported never having had this opportunity. In another study of adult patients undergoing outpatient follow-up,^15^ the majority said they believe that prayers can sometimes influence the healing of their illnesses and that they would like to be asked by their doctors about their beliefs. A small minority had been asked.

The American Academy of Family Medicine (AAFP), which aims to improve and stimulate doctors’ knowledge about the best approach to spiritual issues, establishes strategies for adult patients that, in our opinion, can be adapted to family members and adolescent patients in pediatrics. First, it is reported that the physician’s personal philosophy must always be respected so that there are no major internal conflicts. With this in mind, three questionnaires are proposed:

- The FICA Spiritual Tool, which allows doctors to get to know the spirituality of his or her patient and how much it can affect their life.
- HOPE, which contains specific questions, such as about sources of hope and meaning of life, whether they belong to any religion, and how this may affect their medical care and end-of-life decisions.
- The Open Invite Mnemonic, which addresses patients in a way that induces them to talk more about their spirituality.^16^

Denying the use of religion when caring for patients implies medical negligence, depriving the patient of beneficial care for their treatment, and the possibility of imposing barriers in the doctor-patient relationship. Inadvertently, through ignorance, one can speak or act in ways that generate discomfort and spiritual conflict.^9^

With regard to the acceptance of the dying process and its positive influence on palliative treatment, it is noted that when the participant is religious, it increases his or her chance of considering religious care to be advantageous for the patient. Despite this fact, 95% of the participants believed that having a religion helps patients accept the dying process, and 99% believed that religion relieves the suffering of patients and their family members. This is in agreement with current evidence, which shows that religiosity is related to an overall improvement in the health of the patients and a reduction in anxiety caused by death.^17^ ^,^ ^18^ ^,^ ^19^

Our study had its limitations. The study was performed on the basis of self-assessments, and it is not possible to determine each participant’s knowledge about religions. Additionally, information such as socioeconomic status, nationality and cultural origin were not analyzed. The questionnaire was prepared by the authors of the research, and did not undergo validation or psychometric evaluation.

Positive aspects of the research include the fact that both residents and assistants were considered. Furthermore, the study was carried out in a school hospital, leading us to indirectly observe how pediatric resident doctors that are still in training in the medical field, learn about and value spiritual care.

In conclusion, we noted that although most pediatricians and pediatric residents say they are able to deal with the most prevalent religions in Brazil, affirm that spirituality is beneficial during the dying process and believe that a course on religions should be instituted in Brazilian medical undergraduate programs, little importance is given to the spiritual identities of their patients, which does not give the patient the best dying process.

Finally, we emphasize the understanding that the role of a physician is “to heal at times, to alleviate often, and to comfort always” (unknown author). To do this, we must prepare ourselves to deal with our patients holistically, not just with their physical illnesses.
